# Dysbiotic change in gastric microbiome and its functional implication in gastric carcinogenesis

**DOI:** 10.1038/s41598-022-08288-9

**Published:** 2022-03-11

**Authors:** Jae Yong Park, Hochan Seo, Chil-Sung Kang, Tae-Seop Shin, Jong Won Kim, Joong-Min Park, Jae Gyu Kim, Yoon-Keun Kim

**Affiliations:** 1 Department of Internal Medicine, Chung-Ang University College of Medicine, 102 Heukseok-ro, Dongjak-gu, Seoul, 06973 Republic of Korea; 2 MD Healthcare R&D Institute, World Cup Buk-ro 56-gil, Mapo-gu, Seoul, Republic of Korea; 3 Department of Surgery, Chung-Ang University College of Medicine, Seoul, Republic of Korea

**Keywords:** Cancer, Gastroenterology, Metagenomics

## Abstract

Although there is a growing interest in the role of gastric microbiome on the development of gastric cancer, the exact mechanism is largely unknown. We aimed to investigate the changes of gastric microbiome during gastric carcinogenesis, and to predict the functional potentials of the microbiome involved in the cancer development. The gastric microbiome was analyzed using gastric juice samples from 88 prospectively enrolled patients, who were classified into gastritis, gastric adenoma, or early/advanced gastric cancer group. Differences in microbial diversity and composition were analyzed with 16S rRNA gene profiling, using next-generation sequencing method. Metagenomic biomarkers were selected using logistic regression models, based on relative abundances at genus level. We used Tax4Fun to predict possible functional pathways of gastric microbiome involved in the carcinogenesis. The microbial diversity continuously decreased in its sequential process of gastric carcinogenesis, from gastritis to gastric cancer. The microbial composition was significantly different among the four groups of each disease status, as well as between the cancer group and non-cancer group. Gastritis group was differently enriched with genera *Akkermansia* and *Lachnospiraceae NK4A136 Group*, whereas the cancer group was enriched with *Lactobacillus* and *Veillonella*. Predictive analysis of the functional capacity of the microbiome suggested enrichment or depletion of several functional pathways related to carcinogenesis in the cancer group. There are significant changes in the diversity and composition of gastric microbiome during the gastric carcinogenesis process. Gastric cancer was characterized with microbial dysbiosis, along with functional changes potentially favoring carcinogenesis.

## Introduction

Despite the declining incidence, gastric cancer is still a major health problem as the fourth leading cause of cancer death worldwide^1^. It is widely accepted that chronic inflammatory process commonly precedes the development of gastric cancer, especially in intestinal type cancer^2^. In the initial carcinogenesis process, *Helicobacter pylori* infection plays an important role, leading to subsequent long-lasting chronic inflammation. This pathogen was designated as a class I carcinogen in 1994 by the International Agency for Research on Cancer^3^. However, there seems to be other factors than *H. pylori* infection engaged in the gastric carcinogenesis, considering that only 1–3% of the population infected with *H. pylori* develop gastric cancer, and that even successful eradication treatment does not completely prevent gastric cancer^4,5^.

Improvement of endoscopic devices and techniques made it easier for the researchers to collect gastric juice or tissues. In addition, recent development of molecular biology techniques such as next-generation DNA sequencing has enabled metagenomics research to develop rapidly. Thanks to the remarkable advances in the gastric microbiome research, now we know that gastric microbiota comprises numerous microbes other than *H. pylori*^6^. Recent researches have shown that the composition of gastric microbiome is different between the patients with or without gastric cancer^7,8^. Another study revealed that the composition of gastric microbiota became similar to that of the intestine, when metaplastic change of gastric mucosa was dominant^9^.

In addition to various host and environmental factors, there is a growing interest in the possibility that the change of gastric flora can induce a chronic inflammatory response and precancerous changes, ultimately affecting the gastric carcinogenesis process. However, the majority of previous studies have only revealed the differences in the microbial composition of the stomach, which are insufficient to clarify the role and importance of the microbiome in the process of gastric carcinogenesis.

In this study, we aimed not only to investigate the changes in the composition of gastric microbiome according to each stage of gastric carcinogenesis, but also to interpret the changes in the functional aspect by analyzing metagenomic functions to reveal the impact of microbial changes on gastric cancer development.

## Methods

### Patients and sample collection

Patients aged 19–80 years with gastric adenoma or gastric cancer (neoplasm group), and those with gastritis (gastritis group) were prospectively enrolled from January 2011 to December 2018 at Chung-Ang University Hospital. The inclusion criteria for neoplasm group were patients newly diagnosed with pathologically confirmed gastric cancer or adenoma, whereas that for the gastritis group were patients with gastritis only, without evidence of gastric cancer or adenoma on the endoscopic examination. Patients with a previous history of malignancy including gastric cancer, gastric surgery, taking antibiotics or probiotics within 3 months were excluded. The Vienna classification was used for the diagnosis and classification of gastric neoplasm^10^. In our study, category 3 (non-invasive low-grade adenoma/dysplasia), category 4.1 (high-grade adenoma/dysplasia), and category 4.2 (non-invasive carcinoma [carcinoma in situ]) were all classified as adenoma. Category 4.3 (suspicion of invasive carcinoma) and category 5 (invasive neoplasia) were classified as gastric cancer. Early gastric cancer (EGC) was defined as adenocarcinoma confined to the mucosa or submucosa regardless of regional lymph-node metastasis. Advanced gastric cancer (AGC) was defined as adenocarcinoma infiltrating beyond the submucosal layer. Infection with *H. pylori* was confirmed by the rapid urease test (CLO® test; Kimberly-Clark, UT, USA) or histologic examination with modified Giemsa staining. If either of the two tests showed a positive result, *H. pylori* infection was considered present. This study was approved by the Institutional Review Board of Chung-Ang University Hospital (IRB No. C2016047(1790) & 1772-001-290). All methods in this study were conducted in accordance with the Declaration of Helsinki and informed consent was obtained from all the participants.

Finally, 16 gastritis (GA), 16 gastric adenoma (GAD), 36 EGC, and 20 AGC subjects were enrolled. Gastric juice samples were obtained according to the following protocols. Patients fasted for more than 8 h before the sample collection. In the patients who undergo endoscopic examination or endoscopic resection, a trap tube was connected between the endoscope and the suction tube before the procedure. Gastric juice (7–30 mL) was then collected by suctioning through the endoscope at the beginning of the endoscopic procedure. For the patients who undergo surgical gastrectomy, gastric juice was obtained either via the endoscopic method before the surgery, or during the surgery right after an incision was made in the stomach. To avoid contamination, the endoscopes were washed and disinfected by immersing in a detergent solution containing 7% proteolytic enzymes and 2% glutaraldehyde before use. Immediately after collecting gastric juice, the sample was kept at − 20 °C and immediately moved to the nearby laboratory, without using preservative reagents. Collected gastric juice was diluted to 40 mM by adding 1 M Tris base, and was centrifuged at 600 × g for 10 min at 4 °C to obtain a supernatant. After putting it in a new 15 mL tube, this was centrifuged at 1500 × g for 10 min at 4 °C. The supernatant was then harvested and stored at − 70 °C.

### DNA extraction and sequencing

Before DNA extraction, all samples were incubated in 10 mL of PBS for 24 h after dilution. To separate the bacteria from samples, centrifugation and filtering were performed as elaborated previously^11^. Briefly, bacteria in samples were isolated using centrifugation at 10,000 × g for 10 min at 4 °C. After centrifugation, the pellet, which was comprised of bacteria, was further diluted with 200 μL of PBS to make a suspension. To extract the bacterial DNA, the suspension was boiled for 40 min under 100 °C. Microbial genomic DNA was extracted using a DNeasy PowerSoil kit (QIAGEN, Hilden, Germany) according to the standard protocol provided in the manufacturer’s instructions. Isolated genomic DNA was amplified by targeting the V3–V4 hypervariable regions of 16S rRNA gene using the primers and the amplicon libraries were prepared (primer sequences: 16S_V3_F, 5′-TCGTCGGCAGCGTCAGATGTGTATAAGAGACAGCCTACGGGNGGCWGCAG-3′;

16S_V4_R, 5′-GTCTCGTGGGCTCGGAGATGTGTATAAGAGACAGGACTACHVGGGTATCTAATCC-3′)^11^. For sequencing library reagents, MiSeq Reagent Kit v3 (600-cycle) (Illumina, CA, USA) was used. Nextra XT index Kit v2 Set A (96 Indices, 384 Samples) (Illumina, CA, USA) was used for barcodes and adapters. Library preparation for sequencing followed 16S Metagenomic Sequencing Library Preparation (Part # 15,044,223 Rev. B). All amplicons were sequenced using a MiSeq (Illumina, CA, USA), according to the manufacturer’s instructions. The DNA from bacteria in each sample was quantified by using QIAxpert system (QIAGEN, Hilden, Germany).

### Taxonomic assignment and profiling

Taxonomic assignment was done by the profiling program MDx-Pro ver.2 (MD Healthcare, Seoul, Korea). The representative sequences of the OTUs were finally classified using SILVA 132 database with UCLUST (parallel_assign_taxonmy_uclust.py script on QIIME version 1.9.1) under default parameters. This program used cutadapt for trimming, CASPER for merge, and VSEARCH with de-novo clustering algorithm under a threshold of 97% sequence similarity^12^. To predict possible functional pathways, we used Tax4Fun^13^. Contributions of various OTUs to known biological pathways were calculated based on obtained protein sequences with UProC version 1.2.0 using the Kyoto Encyclopedia of Genes and Genomes (KEGG) Orthology (KO) database for prokaryotes (July 2018 release)^14^.

### Statistical analysis

Significant differences of age, sex, and antral atrophy between groups were determined through one-way analysis of variance, chi-square test, and Fisher’s exact test, respectively. For alpha diversity analysis, samples were rarefied to the minimum read number (1969) to normalize read counts. Principal coordinate analysis (PCoA) was conducted to determine individual taxa-level clustering of groups based on Bray–Curtis dissimilarity distance and weighted UniFrac distance. *p* value for PCoA was calculated by permutational multivariate analysis of variance (PERMANOVA) using dissimilarity matrices. To analyze the difference of microbiome composition and functional pathway between groups, Wilcoxon rank-sum test was performed. Linear discriminate analysis (LDA) effect size (LEfSe) was also used to determine differentially abundant taxa between the groups for selection of biomarkers with statistical and biological significance. When converting the feature table to LEfSe format, 100,000 was used as the normalization value. The LEfSe algorithm utilized Kruskal–Wallis test and LDA with the cut-off LDA score (log10) set as 4. In addition to LEfSe, we also used MaAsLin2 to perform additional analysis of differential abundance between taxa, to show the robustness of the results. For the analysis of relative abundances and functional prediction, Total-Sum Scaling method was used for normalization. Spearman’s rank correlation coefficient was used to analyze correlation between microbiome in gastric juice and functional pathways. *p* value < 0.05 was considered statistically significant. We performed multiple testing correction for the Wilcoxon rank-sum test, using the Benjamini–Hochberg procedure to control the false discovery rate. All analyses were conducted using R Statistical Software (version 3.6.1; R Foundation for Statistical Computing, Vienna, Austria).

## Results

### Demographic and clinical information of the patients

A total of 88 patients were enrolled. The mean age was 61.8 ± 12.2 years, and 64.8% (57/31) were male. The demographic and clinical information of each patients group (GA, GAD, EGC, and AGC) are shown in Table 1. Age and sex did not show significant differences between the four groups (*p* > 0.05).

**Table 1:** Baseline characteristics of enrolled subjects (n = 88).

	Gastritis (n = 16)	Gastric adenoma (n = 16)	EGC (n = 36)	AGC (n = 20)	*p* value
Age (year)	59.8 ± 12.5	65.3 ± 9.6	62.7 ± 10.8	58.8 ± 15.8	0.371^†^
Sex (male/female)	6/10 (37.5%/62.5%)	12/4 (75.0%/25.0%)	25/11 (69.4%/30.6%)	14/6 (70.0%/30.0%)	0.088^‡^
Antral atrophy	15 (93.8%)	16 (100.0%)	34 (94.4%)	20 (100.0%)	0.675^§^

Values are expressed as mean ± standard deviation or number (percentage), unless otherwise specified. Differences between the groups were compared with ^†^one-way analysis of variances, ^‡^chi-square test, or ^§^Fisher’s exact test.

*EGC* Early gastric cancer, *AGC* Advanced gastric cancer.

### Microbiome dysbiosis in gastric cancer

**Figure 1:**
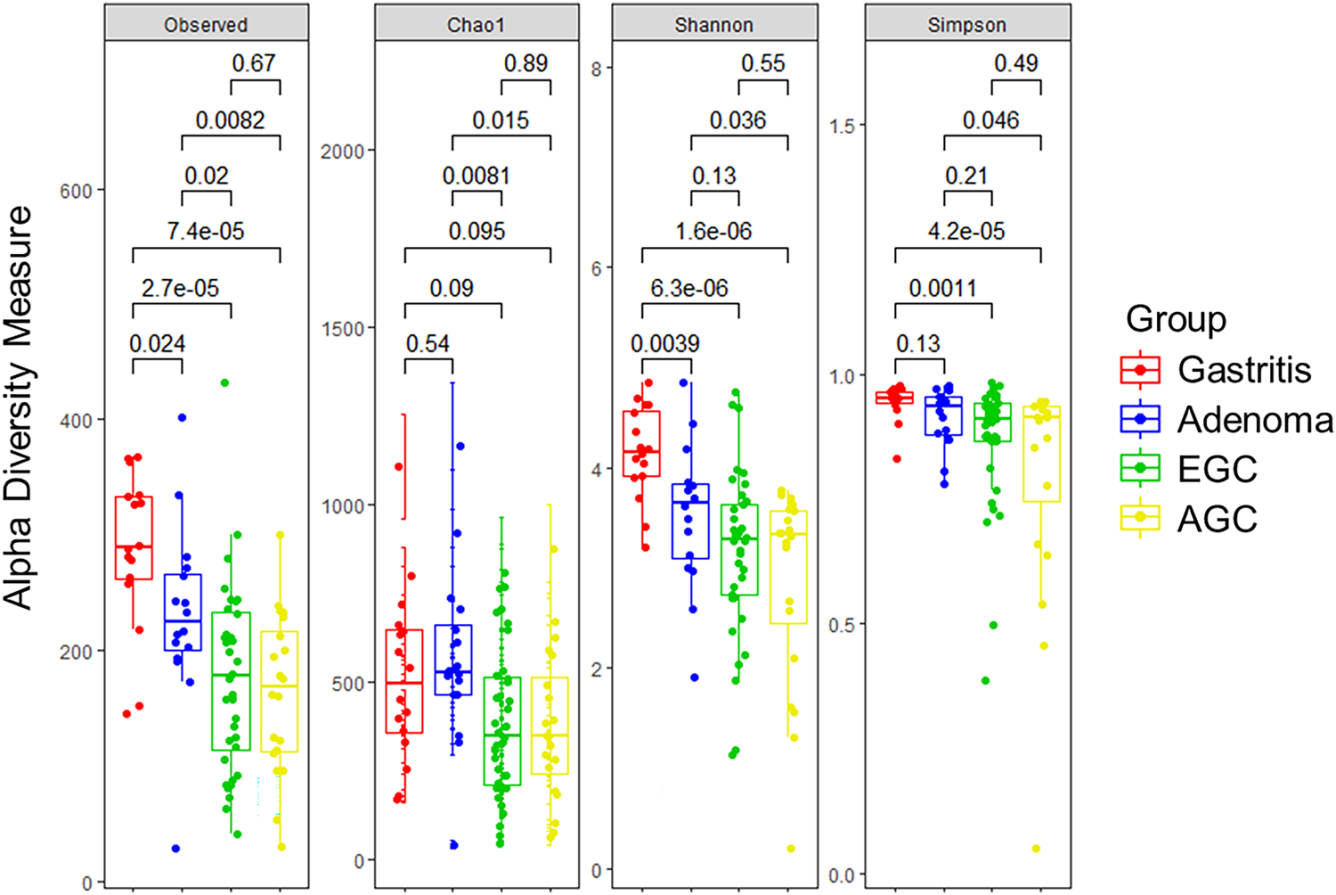
Comparison of alpha diversity of gastric microbiome in each stage of gastric carcinogenesis. Alpha diversity indices (Observed OTUs, Chao1, Shannon, and Simpson index) were calculated and compared between the four groups (GA, GAD, EGC, and AGC).

We measured alpha and beta diversity to investigate the alterations in the microbial composition between the groups, using the gastric juice samples. When we measured the alpha diversity using the number of observed OTUs, it showed a significant and continuous decreasing tendency from GA to GAD (*p* = 0.024), and GAD to EGC (*p* = 0.02). The number of observed OTUs and Shannon index in GA were significantly higher than those in other groups (*p* < 0.05). In addition, Simpson index of GA was significantly higher than that of EGC (*p* = 0.0011) or AGC (*p* < 0.001) (Fig. 1). We additionally compared the alpha diversity between the cancer group (EGC and AGC) and the non-cancer group (GA and GAD), and the former showed significantly lower values than the latter with all alpha diversity measures (Supplementary Fig. S1).

**Figure 2:**
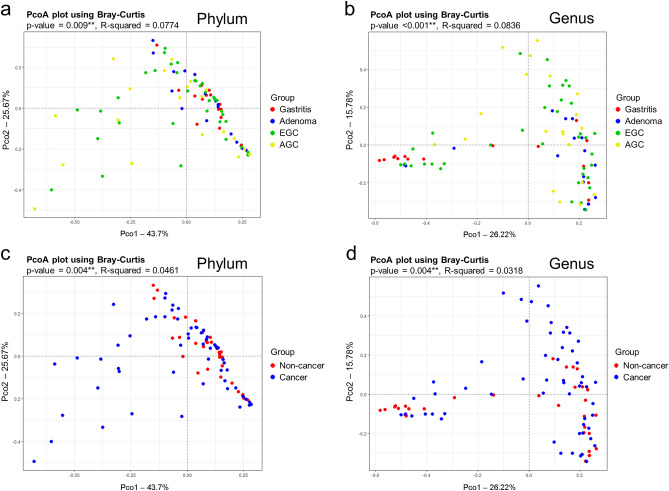
Analysis of beta diversity based on the Bray–Curtis dissimilarity measure and visualization by PCoA plot. The percentage of diversity captured by each coordinate is indicated in the axis. The microbial compositions were compared between the four groups (GA, GAD, EGC, and AGC) (**a**) at the phylum level and (**b**) at the genus level. The microbial compositions were also compared between the cancer group (EGC and AGC) and non-cancer group (GA and GAD) (**c**) at the phylum level and (**d**) at the genus level.

To calculate the beta diversity, samples were plotted along the two principal coordinates (PCo1 and PCo2) based on Bray–Curtis dissimilarity for evaluating the cluster of groups. There was a significant difference in the microbial composition among the four patient groups, at both the phylum and the genus levels (PERMANOVA R^2^ = 0.0774, *p* = 0.009; and R^2^ = 0.0836, *p* < 0.001, respectively; Fig. 2a,b). The total diversities explained by the top two principal coordinates were 69.4% in the phylum level and 42.0% in the genus level. When we compared the microbial composition between the cancer group and the non-cancer group, the difference was still significant at both the phylum and the genus levels (PERMANOVA R^2^ = 0.0461, *p* = 0.004; and R^2^ = 0.0318, *p* = 0.004, respectively; Fig. 2c,d). The significant difference in the microbial composition was also evident when analyzed with the weighted UniFrac distance metric (Supplementary Fig. S2). To investigate if different sample collection methods (endoscopy or surgery) affected the microbial composition, we analyzed the beta-diversity between the endoscopy group and the surgery group in EGC and AGC patients respectively. There were no significant differences in the microbial compositions according to the sampling methods in each group of patients (Supplementary Fig. S3).

### Different microbial compositions between groups and development of biomarkers

**Figure 3:**
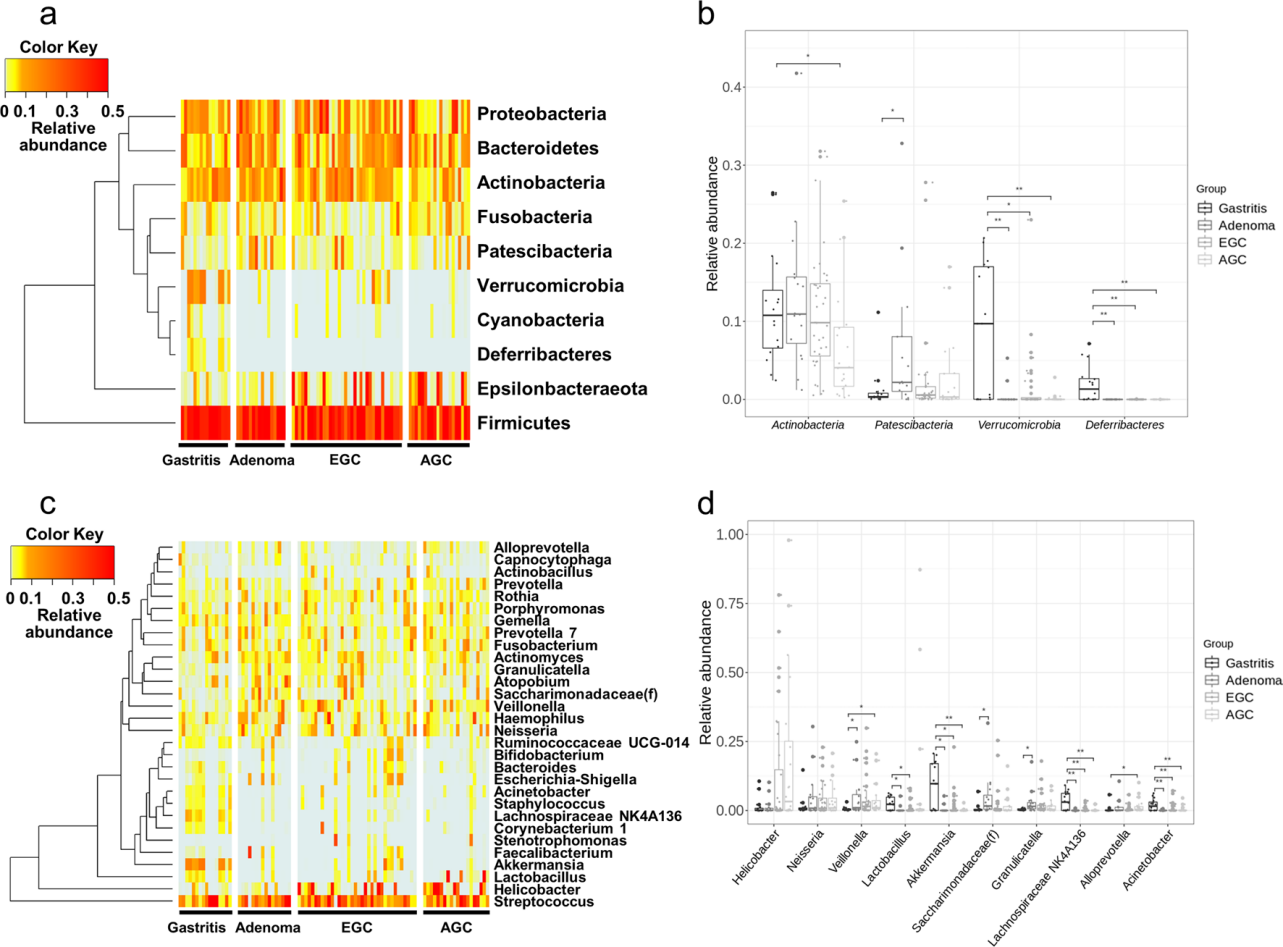
Differences in the relative abundance of gastric microbiome between the disease groups at the phylum and genus level. (**a**) A heatmap demonstrating the relative abundances of gastric microbiome at the phylum level. (**b**) The relative abundances of the bacteria were compared at the phylum level between the four groups (GA, GAD, EGC, and AGC), and those showing clinical significance were demonstrated. (**c**) A heatmap demonstrating the relative abundances of gastric microbiome at the genus level. (**d**) The relative abundances of the bacteria were compared at the genus level between the four groups (GA, GAD, EGC, and AGC), and those showing clinical significance were demonstrated. *q < 0.05, ** < 0.01.

Relative abundance of gastric microbiome was differed by disease (Supplementary Tables 1, 2). At the phylum level, *Firmicutes* was the most dominant taxa without significant difference between the groups (Fig. 3a). *Verrucomicrobia* and *Deferribacteres* were significantly more abundant in GA than other groups (q < 0.05), whereas *Actinobacteria* were significantly less abundant in AGC than GA group (q < 0.05). In addition, the relative abundance of *Patescibacteria* in GAD was significantly higher than that in GA (q < 0.05) (Fig. 3b). At the genus level, *Streptococcus* was the most dominant taxa regardless of disease status. There were 5 patients whose gastric microbiota was dominated (> 50%) by *Helicobacter*, who were all from the cancer group (Fig. 3c). The relative abundance of *Helicobacter* was 10.0-fold higher in the cancer group than in the non-cancer group (q < 0.01), showing the highest value especially in AGC. The relative abundances of *Akkermansia, Lachnospiraceae NK4A136*, and *Acinetobacter* were significantly higher in GA than other groups (q < 0.05), and that of *Lactobacillus* was significantly higher in GA group than GAD or EGC groups (q < 0.05). In addition, *Saccharimonadaceae(f)* was significantly more abundant in GAD than other groups (q < 0.05). *Granulicatella* was also overabundant in GAD than other groups, but clinical significance was present only when compared to GA (q < 0.05). On the other hand, the relative abundance of *Veillonella* was significantly lower in GA than GAD or AGC groups (q < 0.05), and that of *Alloprevotella* was significantly lower in GA than AGC group (q < 0.05) (Fig. 3d).

**Figure 4:**
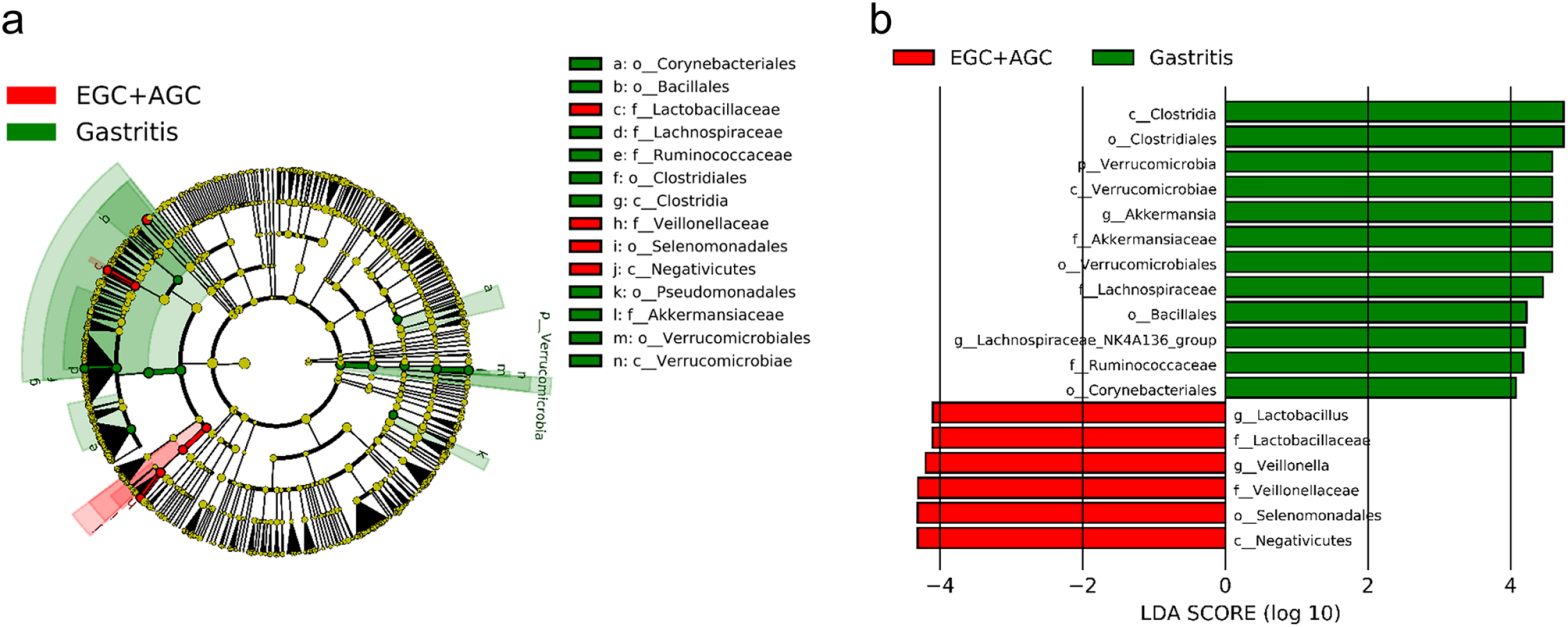
Identification of microbial biomarkers for gastric cancer with LEfSe. (**a**) Cladogram showing differentially abundant taxa of the gastric microbiome between the cancer group and gastritis group. (**b**) Association of specific microbial taxa with the cancer group and gastritis group was investigated with linear discriminant analysis (LDA) effect size (LEfSe), using a LDA cut-off score of 4.0 or greater. Taxa enriched in the cancer group are indicated in red, and taxa enriched in gastritis group are indicated in green.

To identify metagenomic biomarkers of gastric cancer, we used LEfSe to analyze differentially abundant microbial taxa between the cancer group and GA. Among the metagenomics biomarker candidates, 18 taxa showing significant differences in the relative abundance with LDA score > 4 were finally selected: 6 taxa increased, 12 taxa decreased in the cancer group (Fig. 4a,b). In the gastric juice samples, *Corynebacteriales* order, *Ruminococcaceae* family, *Bacillales* order, *Lachnospiraceae* family, *Verrucomicrobiales* order, *Akkermansiaceae* family, and *Clostridiales* order were enriched in GA, while *Lactobacillaceae* family, *Veillonellaceae* family, and *Selenomonadales* order were enriched in the cancer group. *Clostridiales* order in GA and *Selenomonadales* order in the cancer group showed the highest LDA score of 4.74 and 4.30, respectively. At the genus level, microbiota of GA was differently enriched with genera *Akkermansia* and *Lachnospiraceae NK4A136 Group,* whereas the cancer group was enriched with *Lactobacillus* and *Veillonella.* The MaAsLin2 analysis result was also similar to the LEfSe result, indicating the robustness of biomarker analysis. Detailed data are added as Supplementary Data 1.

### Functional analysis of metagenome

**Figure 5:**
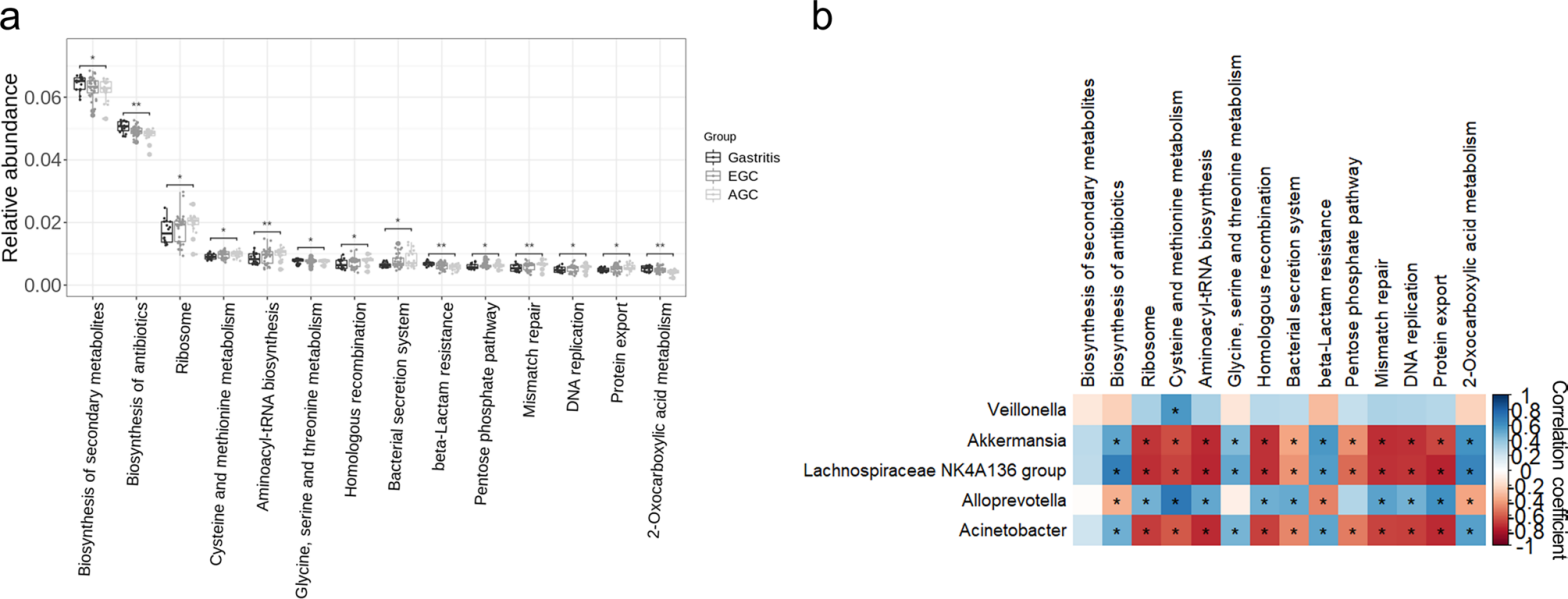
Predicted functional compositions of the gastric microbiome using Tax4fun based on KEGG. (**a**) Difference of predicted functional pathways between GA, EGC, and AGC groups. (**b**) Correlation between the predicted functional pathways and microbial biomarkers for gastric cancer are shown.

To predict the functional potentials of the microbiomes based on 16S rDNA sequences, we performed Tax4Fun analysis based on KOs. Dominant functional pathways were identified, and changes in the functional capacity of the microbiome were analyzed between the GA and cancer group (Supplementary Table 3). Among the dominant pathways, only the Biosynthesis of antibiotics (ko01130) and beta-Lactam resistance (ko01501) showed significant differences between GA and the cancer group. When EGC and AGC were analyzed separately to find out functional pathways significantly different from GA group, there were none in the EGC group, whereas 14 pathways were identified in the AGC group (Supplementary Table 4). The functional changes in the microbiome of AGC group was characterized by significantly increased representation of predicted KEGG pathways: Ribosome (ko03010), Cysteine and methionine metabolism (ko00270), Aminoacyl-tRNA biosynthesis (ko00970), Homologous recombination (ko03440), Bacterial secretion system (ko03070), Pentose phosphate pathway (ko00030), Mismatch repair (ko03430), DNA replication (ko03030), and Protein export (ko03060) (q < 0.05). On the other hand, pathways related to Biosynthesis of secondary metabolites (ko01110), Biosynthesis of antibiotics (ko01130), Glycine, serine and threonine metabolism (ko00260), beta-Lactam resistance (ko01501), and 2-Oxocarboxylic acid metabolism (ko01210) were significantly enriched in GA (q < 0.05, Fig. 5a).

To find out the relationship between the microbial changes and the functional differences during the carcinogenesis process, the correlation between the differently enriched bacterial genera and the significantly different functional pathways between GA and the AGC group were analyzed. *Akkermansia*, *Lachnospiraceae NK4A136 group,* and *Acientobacter* showed a highly negative correlation (r <  − 0.4) with Ribosome (ko03010), Cysteine and methionine metabolism (ko00270), Aminoacyl-tRNA biosynthesis (ko00970), Homologous recombination (ko03440), Pentose phosphate pathway (ko00030), Mismatch repair (ko03430), DNA replication (ko03030), and Protein export (ko03060), and a highly positive correlation (r > 0.4) with Biosynthesis of antibiotics(ko01130), beta-Lactam resistance (ko01501), and 2-Oxocarboxylic acid metabolism (ko01210). While these three taxa showed very similar functional correlation profiles to each other, *Veillonella* and *Alloprevotella* both presented, interestingly, an exact opposite trend of functional correlation compared to the three taxa (Fig. 5b).

## Discussion

In this study, we identified the changes of gastric microbiome during the process of gastric carcinogenesis, and tried to investigate the functional potentials of the microbiome involved in the process. We have demonstrated that the composition of gastric microbiome in patients with gastric cancer was significantly different from that of patients without gastric cancer. The gastric cancer dysbiosis enabled us to identify metagenomic biomarkers for gastric cancer, and functional pathways correlated with these biomarkers were further analyzed to elucidate the possible role of microbiome in the gastric carcinogenesis.

In our study, the alpha diversity of gastric microbiome showed continuously decreasing tendency in its sequential process of gastric carcinogenesis, from gastritis to gastric cancer. In addition, the microbial composition was significantly different among the four groups of each disease status (GA, GAD, EGC, and AGC), which was still evident when compared between the cancer group and non-cancer group. It is now well known that reduced microbial diversity is related to a diseased status in numerous disorders, comprising not only gastrointestinal, but also extragastrointestinal or systemic diseases^15^. Microbial dysbiosis can stimulate aberrant proinflammatory immune responses and increase susceptibility to invading pathogens, which can consequently lead to disease processes^16^. This possible pathogenic role of microbiome-host interaction has been inferred in various diseases such as colon cancer, inflammatory bowel disease (IBD), dementia, diabetes, etc^17^. Previous studies have shown that this finding is also evident in gastric malignancy, reporting decreased microbial diversity in gastric cancer^18–20^.

The compositional analysis of microbiome has revealed that the microbiome of gastric juice was dominated by *Firmicutes*, *Proteobacteria*, *Bacteroidetes*, and *Actinobacteria* in phylum level, as repeatedly shown in previous studies^21,22^. In gastric cancer group, there were changes in the microbial composition, characterized by decreased abundance of *Verrucomicrobia* and *Deferribacteres*. We further analyzed specific compositional differences of gastric microbiome between the gastritis group and the cancer group using LEfSe, to identify metagenomic biomarkers for gastric cancer. In genus level, *Akkermansia* and *Lachnospiraceae NK4A136 Group* were significantly overabundant in gastritis group. Several reports suggested that *Akkermansia*, which belongs to the phylum *Verrucomicrobia*, affects intestinal immunity as well as glucose and lipid metabolism^23,24^. *Akkermansia* can synthesize glycosidase to degrade mucin, a glycosylated protein which promote the barrier function of the gastrointestinal tract, and help maintain the balance of mucin and short chain fatty acids^23^. Its abundance in the human intestinal tract is inversely correlated with several disease states such as obesity and glucose intolerance. Previous studies have also shown that the abundance of *Akkermansia* was decreased in inflammatory status of colon, such as IBD or acute appendicitis^25–27^. These diseases might be closely related to the integrity or thickness of the intestinal mucus layer. Although the mucus layer of colon is considered as the optimal ecological niche for *Akkermansia*, they are also found in other gastrointestinal regions including the oral cavity, pancreas, biliary system, and small intestine^25^. A recent animal study also observed that progression to gastric cancer is associated with depletion of *Akkermansia*, which is consistent with the results from the present study^28^. Therefore, we can possibly infer that *Akkermansia* might have a protective role against gastric carcinogenesis by influencing the integrity of superficial gastric layer, as accumulated damage and atrophy of the gastric mucosa accompanied by chronic inflammation is important in the process of gastric cancer development. The relative abundance of *Lachnospiraceae NK4A136 group,* a butyrate-producing bacterium, was also decreased in gastric cancer in our study. A study on mice observed that *Lachnospiraceae NK4A136 group* in the cecal contents was positively correlated with the level of acetic acid and butyric acid, while this genus was negatively correlated with obesity-related indicators such as serum cholesterol and aminotransferase levels at the same time^29^. Previous studies repeatedly showed that *Lachnospiraceae NK4A136 group* can produce short-chain fatty acids such as acetic acid or butyric acid, which seems to have an important role in the diet-gut microbiome-host metabolism axis^30–32^. Studies with IBD animal models revealed that *Lachnospiraceae NK4A136 group* was decreased in these disease models^32,33^. There are also some reports revealing that this taxon was decreased in the feces of IBD patients, as well as patients with dementia^34,35^. Increased gut permeability with reduced abundance of *Lachnospiraceae NK4A136* was evident in these patients^34^. Recent studies even showed that this taxon is closely related to gut barrier function, and the barrier function could be improved by increasing *Lachnospiraceae NK4A136 group* in mice^36–38^. Although these findings are mainly observed from studies with fecal samples and colonic diseases, these bacteria have an important role in maintaining the integrity of superficial layer of gastrointestinal tract. Therefore, it could be inferred that decreased abundance of *Akkermansia* and *Lachnospiraceae NK4A136 group* in gastric cancer might be related to the damaged integrity of superficial gastric layer, making the gastric mucosa more vulnerable to chronic inflammation and malignant change.

On the other hand, we found that *Lactobacillus* and *Veillonella* were significantly more abundant genera in gastric cancer. *Lactobacillus*, a taxonomically complex group of bacteria comprising more than 260 species, is part of commensal microflora of human gastrointestinal tract. However, some *Lactobacillus* species may be related to malignant diseases, represented by gastric cancer. Increase in the abundance of *Lactobacillus*, which is a lactic acid bacterium as well as a probiotic bacterium, has been consistently observed in human and mice with intestinal metaplasia or gastric cancer^18,28,39,40^. Along with these findings, there are some experimental evidences revealing that commensal intestinal bacteria could promote the development of gastric cancer. Considering this, increased abundance of *Lactobacillus* in atrophic stomach might suggest a role for gastric colonization with intestinal bacteria in the progression of gastric cancer. *Veillonella* is one of the most predominant oral microbes and early colonizers. This bacterium serves as a bridging species to provide an environment for downstream pathogens related to gastrointestinal cancers^41,42^. Some studies have previously shown that the abundance of *Veillonella* is increased in the gut of patients with gastric cancer, which has a good diagnostic value in distinguishing gastric cancer from the control^43,44^. In gastric cancer patients, this bacterium also seems to have an important role as a network-hub in microbial community alteration in the gut after gastrectomy^45^. In addition, as a nitrate-reducing bacterium, *Veillonella* catalyzes the nitrite production from the nitrate reduction^46^. This bacterium could be responsible for the accumulation of nitrite in the stomach, which is a precursor of the endogenous N-nitroso compounds. Previous studies have shown that a large amount of nitrite was detected in an environment where nitrate-reducing bacteria other than *H. pylori*, such as *Veillonella*, were mainly present^47^. As N-nitroso compounds have a crucial role in the development of gastric cancer, we can assume that *Veillonella* may affect the carcinogenesis process with its nitrate-reducing function. There was a tendency that *Helicobacter* was generally enriched in gastric cancer, although there were differences according to the patient grouping. Although the exact mechanism by which *H. pylori* causes gastric cancer is largely unknown, initiation of chronic inflammatory process and direct toxic effect of virulence factors from the pathogen itself are recognized as the main mechanisms^48^. Infection with *H. pylori* greatly influences the composition of gastric flora and induces microbial dysbiosis. Premalignant mucosal changes including atrophy and intestinal metaplasia after *H. pylori* infection lead to environmental changes in the stomach represented by decreased gastric acid secretion. Eventually, the proportion occupied by gastric flora tends to be replaced by other oral or intestinal bacteria^8,9,18^.

We further explored the functional potential of gastric microbiome changes, to get a glimpse of the mechanism of host-microbiome interaction in the process of gastric carcinogenesis. We observed some changes in the predicted functional pathways which may be related to the compositional differences of microbiome. These changes included several pathways associated with amino acid metabolism in gastric cancer. The influence of microbiome on systemic or cellular metabolism has recently emerged as a hot issue in cancer research. In the process of tumor progression, microbiome metabolites can regulate inflammation, proliferation, and cell death by modulating the tumor microenvironment^49^. In our study, the cysteine and methionine metabolism pathway was significantly enriched in the AGC group. Cysteine and methionine, which are sulfur-containing amino acids, are important for cancer cell growth and metabolism^50,51^. Hydrogen sulfide can be formed by bacteria using these sulfur-containing amino acids, which can inhibit butyrate metabolism, consequently leading to mucosal damage and inflammation of the intestinal tract^52^. In colorectal cancer, microbe-derived hydrogen sulfide seems to play a role in cancer progression and colon health^53^. Similarly, enrichment of cysteine and methionine metabolism pathway in gastric cancer is predictive of increased metabolism of these amino acids by gastric microbiome, which might eventually lead to inflammation of gastric mucosa. Chronic inflammation of gastric epithelium is the essential step of gastric carcinogenesis, as inflammation of the tissue around the tumor can cause field cancerization by promoting genetic instability and inducing mutations^54^.

In the meanwhile, there is another prerequisite for chronic inflammation to persist. To maximize the pro-inflammatory and possible carcinogenic effect of pathogens, these microbes should survive and thrive against the harsh environment in the stomach. Consequently, we can easily anticipate that the DNA replication or repair function will be increased in response to this hostile situation, which were the findings in our study. Recently, another research group has also demonstrated that the gastric cancer-related changes in microbiome functionality included a significant increase in genetic material associated with replication and repair, and translation^55^. In fact, similar findings have been reported in the analysis of association between colorectal cancer and fecal microbiota^56^. In our study, pathways related to metagenomics functions involving ribosome, aminoacyl-tRNA biosynthesis, homologous recombination (HR), mismatch repair, and DNA replication were enriched in AGC group compared to gastritis group. Aminoacyl-tRNAs not only play a major role in protein biosynthesis, which is essential for the survival and growth of all cells, but are also involved in several other important reactions. Aminoacyl-tRNAs can affect the structure of cell envelope by the aminoacylation of phospholipids in the cell membrane and by linking the peptidoglycan in the cell walls of pathogens. These important roles of aminoacyl-tRNA can affect the interaction between the bacteria and antimicrobial peptides^57,58^. Some even suggests that inhibition of the enzymes related to aminoacyl-tRNA might increase the sensitivity of bacterial pathogens to the antibiotics or host innate immune system^59^. This suggests that aminoacyl-tRNA is important for the growth and survival of cancer-associated pathogens against the host immune system. HR is a major DNA repair mechanism in bacteria, and facilitates the incorporation of genetic material received from a donor cell via horizontal gene transfer and transformation. This is also how bacteria cope with the environmental stress and expand the genetic diversity by exchanging genetic material between and within species^60^. Enrichment of DNA replication-related pathway in AGC can also be interpreted in a similar context of bacterial thriving and survival. Therefore, our observation of changes in the functional pathways in gastric cancer may infer the role of gastric microbiome in the carcinogenesis process.

Recently, contamination has emerged as an issue in microbiome analysis in hospital setting, and caution is required especially in a low biomass environment. It is known that use of control sample can be helpful to minimize the effect of contamination^61^. Although the contamination effect by the hospital microbiome cannot be completely excluded in our study, this contamination effect does not seem to be high, as all the samples were collected from the outpatient setting or were obtained the very next day after admission. Gastric juice was also collected using endoscopes which were handled following the endoscopic disinfection/cleaning/storage management guidelines thoroughly, or under an aseptic environment in the operation room. In addition, it is difficult to regard gastric juice as a low biomass sample. Although a negative control sample was not used, which is a limitation, we performed NGS analysis using a mock community consisting of 6 known strains for every plate (128 samples) with the same kit and reagent, as a positive control, to minimize bias due to contamination.

Our study has some limitations. We only used gastric juice samples to analyze the gastric microbiome. There could be some discrepancies in the microbial composition between gastric juice and mucosal tissues, and bystander bacteria can be included in the gastric juice. Further studies should be performed with both samples, to find out the association of microbial composition between the sample types and to verify cancer-related microbial changes more clearly. Another limitation was that although we showed significant changes of gastric microbiome in the carcinogenesis process, this does not necessarily indicate that the microbial change in gastric cancer is the key causative factor in the gastric carcinogenesis. Nevertheless, we performed additional functional analysis to further elucidate the possible role of gastric microbiome in carcinogenesis, and suggested some logical explanations for the host-microbiome interaction, although the effect sizes of the functional differences were rather small. Still, this indirect functional study was not sufficient to reveal the functional implication of microbiome and its causal relationship with gastric carcinogenesis. For this purpose, multi-omics studies including metabolomics or metatranscriptomics, along with metagenomics, are needed. We were also unable to compare the patient groups with a completely symptom-free normal control group. Actually, it is very hard to get gastric juice samples from normal subjects without any symptom. Instead, we tried to include gastritis patients without any evidence of gastric neoplasm, which had a role of non-neoplastic control group. Despite these limitations, we prospectively enrolled relatively large number of subjects including control gastritis group and patients representing each stage of gastric carcinogenesis process. Our findings successfully showed distinct microbial changes in the carcinogenesis process with their functional implications.

In conclusion, our study identified significant changes in the diversity and composition of gastric microbiome during the gastric carcinogenesis process. Microbial dysbiosis worsened as the disease progressed from gastritis to the precancerous stages and gastric cancer. We demonstrated that there were distinct changes of relative abundance of specific taxa in gastric cancer compared to gastritis. Subsequent analysis of metagenomics biomarkers in functional aspect suggests that gastric microbiome has potential roles in the process of gastric carcinogenesis. These findings would help us understand the pathogenesis of gastric cancer in the aspect of microbiome, which could hopefully give us some insight on gastric cancer prevention and treatment in the future.

## Supplementary Information

Supplementary Information 1.

Supplementary Information 2.

Supplementary Information 3.

Supplementary Information 4.

Supplementary Information 5.

Supplementary Information 6.

Supplementary Information 7.

Supplementary Information 8.

## Data Availability

All data generated or analyzed during this study are included in this published article (and its Supplementary Information files). Metagenomic sequencing reads can be accessed from National Center for Biotechnology Information (NCBI) BioProject accession ID PRJNA794918.
