# Supplementary figures and images for: Dysbiotic change in gastric microbiome and its functional implication in gastric carcinogenesis
Source: Sci Rep. 2022 Mar 11;12:4285. doi: 10.1038/s41598-022-08288-9 (PMC8917121; doi:10.1038/s41598-022-08288-9)

## Slide 1
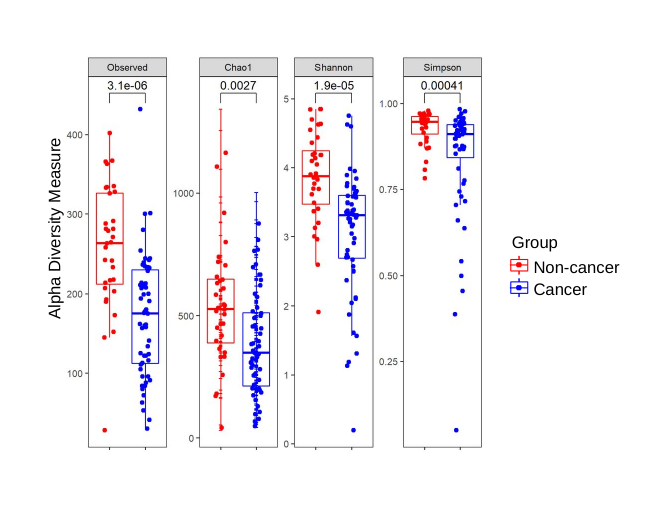

Alpha Diversity Measure
Group
Non-cancer
Cancer

Supplement: Supplementary file 2 — Supplementary Information 2. [file 41598_2022_8288_MOESM2_ESM.pptx]

## Slide 1
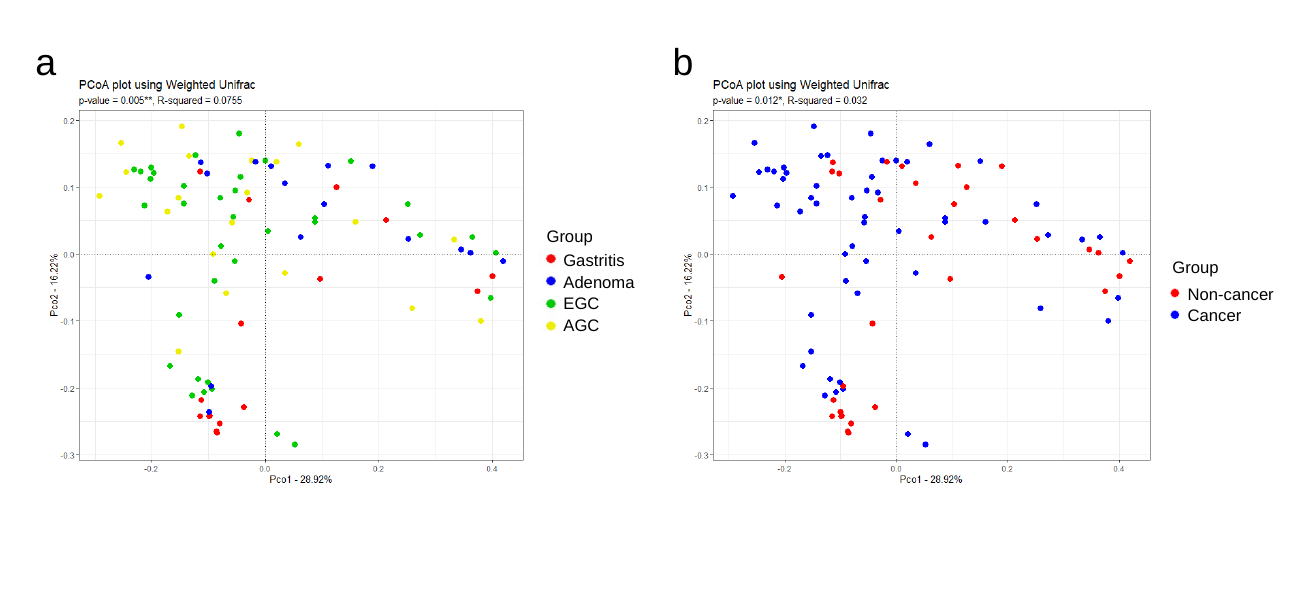

a
b
Group
Gastritis
Adenoma
EGC
AGC
Group
Non-cancer
Cancer

Supplement: Supplementary file 3 — Supplementary Information 3. [file 41598_2022_8288_MOESM3_ESM.pptx]

## Slide 1
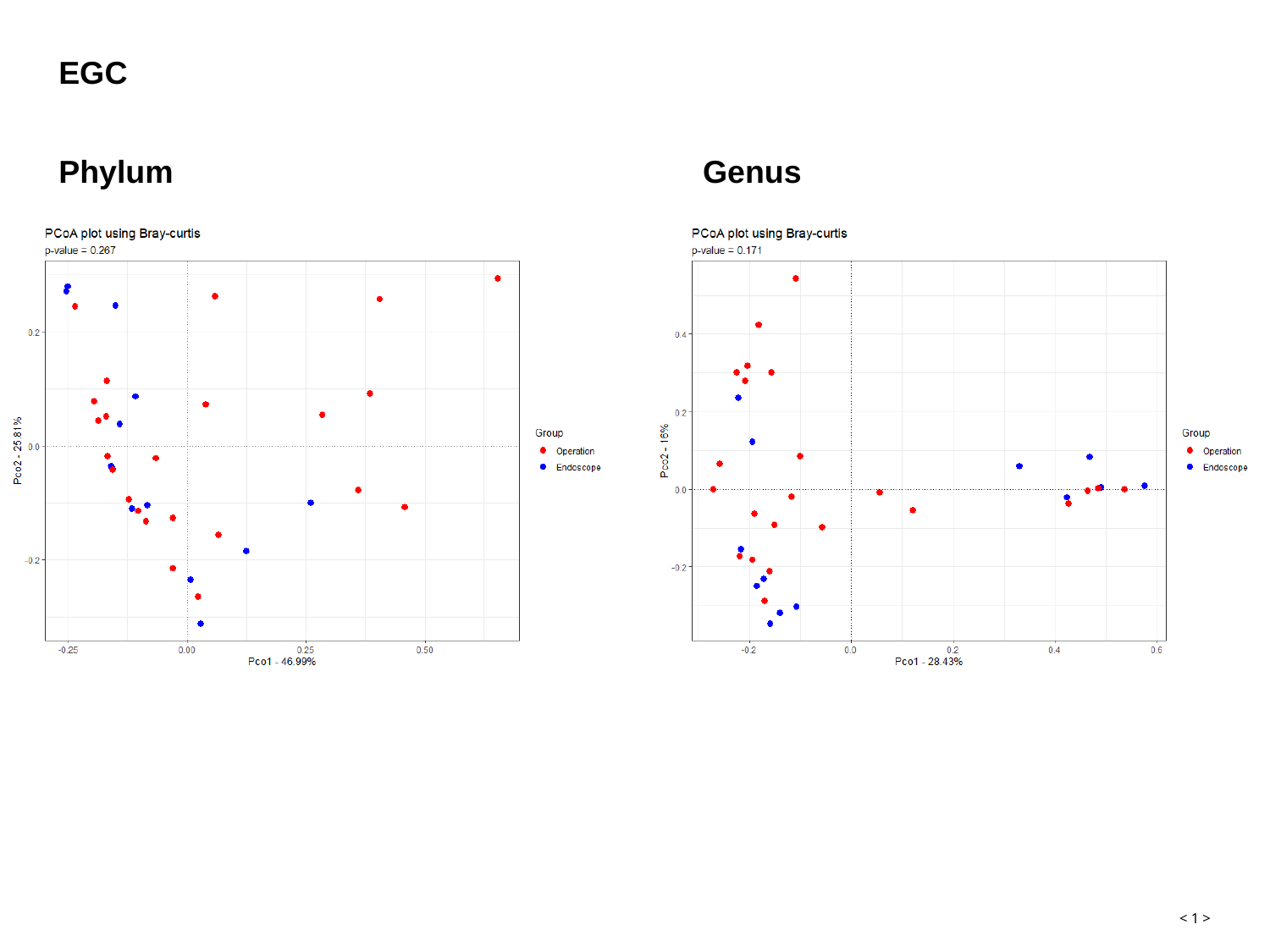

EGC
Phylum
 Genus

## Slide 2
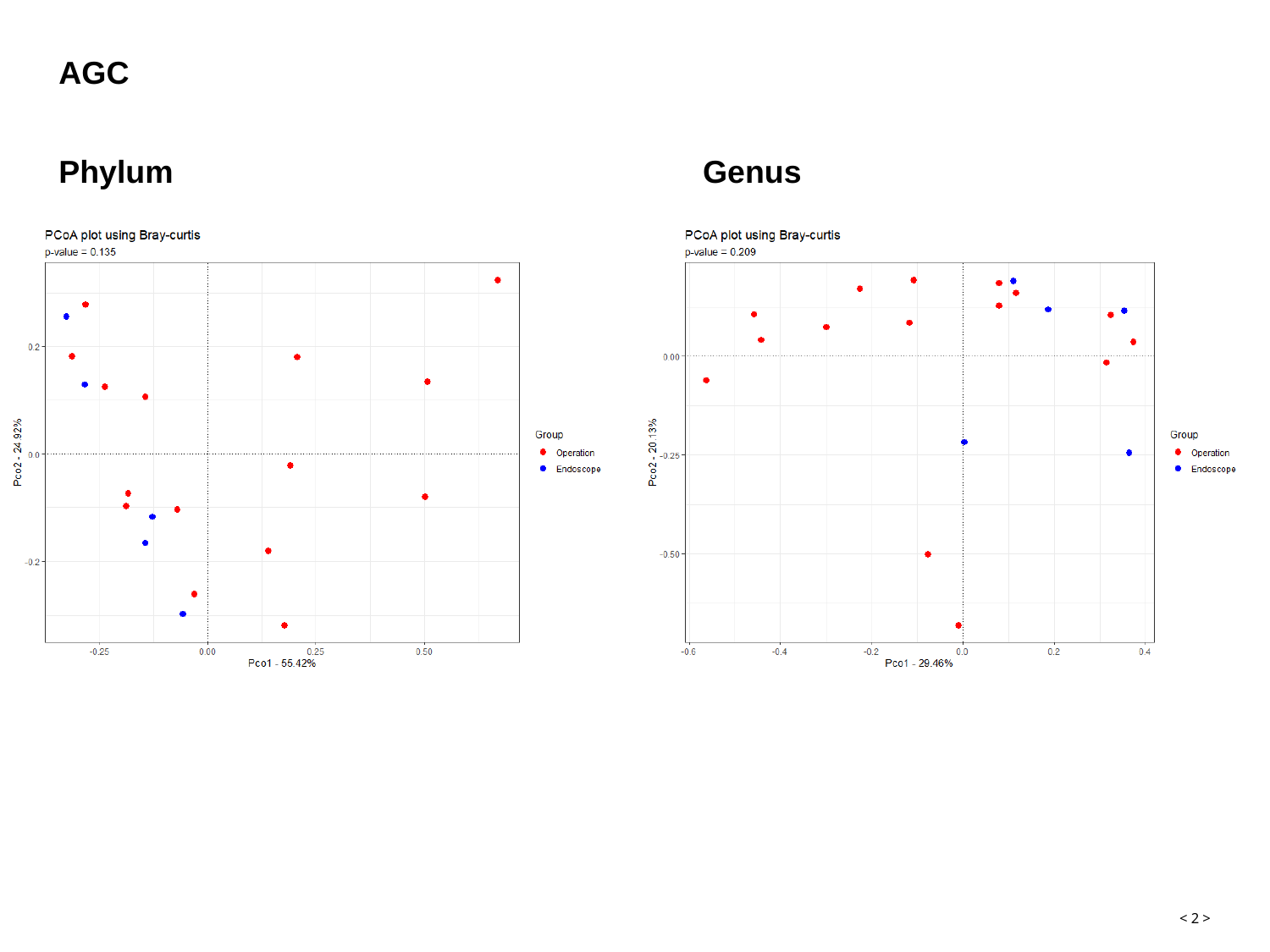

AGC
Phylum
 Genus

Supplement: Supplementary file 4 — Supplementary Information 4. [file 41598_2022_8288_MOESM4_ESM.pptx]
