# Supplementary material for: Dysbiotic change in gastric microbiome and its functional implication in gastric carcinogenesis
Source: Sci Rep. 2022 Mar 11;12:4285. doi: 10.1038/s41598-022-08288-9 (PMC8917121; doi:10.1038/s41598-022-08288-9)
Supplement: Supplementary file 5 — Supplementary Information 5. [file 41598_2022_8288_MOESM5_ESM.doc]

**Supplementary Table 1. Relative abundance of gastric microbiome at phylum level**

|  | **GA** | **GAD** | **EGC** | **AGC** | **q-value^†^** | | | | | |
| --- | --- | --- | --- | --- | --- | --- | --- | --- | --- | --- |
| **Taxonomy** | **Mean ± SE** | **Mean ± SE** | **Mean ± SE** | **Mean ± SE** | **GA-GAD** | **GA-EGC** | **GA-AGC** | **GAD-EGC** | **GAD-AGC** | **EGC-AGC** |
| *Firmicutes* | 0.4831±0.0285 | 0.4666±0.0373 | 0.4208±0.0294 | 0.4204±0.0549 | 0.9852 | 0.5583 | 0.4137 | 0.6840 | 0.5189 | 0.8721 |
| *Proteobacteria* | 0.1185±0.0209 | 0.1421±0.0299 | 0.1465±0.0212 | 0.1181±0.0321 | 0.9852 | 0.7760 | 0.5189 | 0.9141 | 0.5189 | 0.6973 |
| *Bacteroidetes* | 0.0951±0.0248 | 0.1333±0.0275 | 0.1182±0.0158 | 0.1173±0.0235 | 0.9852 | 0.5583 | 0.7550 | 0.8227 | 0.7550 | 0.8721 |
| *Actinobacteria* | 0.1177±0.0182 | 0.1312±0.0243 | 0.1121±0.0131 | 0.0694±0.0167 | 0.9852 | 0.7760 | **0.0446*** | 0.6840 | 0.1471 | 0.1524 |
| *Epsilonbacteraeota* | 0.0162±0.0079 | 0.0157±0.0073 | 0.1196±0.0351 | 0.1877±0.064 | 0.9852 | 0.5583 | 0.1857 | 0.6840 | 0.1741 | 0.6973 |
| *Fusobacteria* | 0.0293±0.0094 | 0.0313±0.0103 | 0.0269±0.0088 | 0.0437±0.0117 | 0.9852 | 0.6509 | 0.4137 | 0.6840 | 0.5189 | 0.1921 |
| *Patescibacteria* | 0.0120±0.0068 | 0.0624±0.0220 | 0.0247±0.0102 | 0.0273±0.0109 | **0.0429*** | 0.5583 | 0.9365 | 0.1254 | 0.1741 | 0.8721 |
| *Verrucomicrobia* | 0.0873±0.0211 | 0.0048±0.0035 | 0.0151±0.0070 | 0.0018±0.0014 | **0.0077**** | **0.0131*** | **0.0033**** | 0.6840 | 0.9442 | 0.5739 |
| *Deferribacteres* | 0.0194±0.0059 | 0 | < 0.0001 | < 0.0001 | **0.0003**** | **0.0000**** | **0.0001**** | 0.6840 | 0.5189 | 0.8721 |

^†^ Differences between groups were compared with Mann-Whitney U test with Benjamini & Hochberg correction. *q < 0.05; **< 0.01.
