# Supplementary material for: Dysbiotic change in gastric microbiome and its functional implication in gastric carcinogenesis
Source: Sci Rep. 2022 Mar 11;12:4285. doi: 10.1038/s41598-022-08288-9 (PMC8917121; doi:10.1038/s41598-022-08288-9)
Supplement: Supplementary file 6 — Supplementary Information 6. [file 41598_2022_8288_MOESM6_ESM.doc]

**Supplementary Table 2. Relative abundance of gastric microbiome at genus level**

|  | **GA** | **GAD** | **EGC** | **AGC** | **q-value^†^** | | | | | |
| --- | --- | --- | --- | --- | --- | --- | --- | --- | --- | --- |
| **Taxonomy** | **Mean ± SE** | **Mean ± SE** | **Mean ± SE** | **Mean ± SE** | **GA-GAD** | **GA-EGC** | **GA-AGC** | **GAD-EGC** | **GAD-AGC** | **EGC-AGC** |
| *Streptococcus* | 0.1955±0.0519 | 0.2248±0.0394 | 0.2007±0.0246 | 0.1969±0.0391 | 0.7484 | 0.7477 | 0.9527 | 0.9546 | 0.7537 | 0.8509 |
| *Helicobacter* | 0.0145±0.0078 | 0.0139±0.0074 | 0.1176±0.0352 | 0.1861±0.0642 | 0.9154 | 0.6790 | 0.2331 | 0.6621 | 0.2038 | 0.7207 |
| *Haemophilus* | 0.0245±0.0099 | 0.0515±0.0017 | 0.0371±0.0127 | 0.0357±0.017 | 0.8898 | 0.7848 | 0.9527 | 0.6621 | 0.7537 | 0.8509 |
| *Neisseria* | 0.0156±0.0089 | 0.0496±0.0212 | 0.0381±0.0098 | 0.0408±0.0139 | 0.8898 | 0.7477 | 0.8935 | 0.9546 | 1.0000 | 0.9250 |
| *Veillonella* | 0.0054±0.0020 | 0.0461±0.018 | 0.0429±0.0118 | 0.0433±0.0150 | **0.0377*** | 0.0562 | **0.0338*** | 0.9546 | 1.0000 | 0.9250 |
| *Lactobacillus* | 0.0238±0.0061 | 0.0044±0.0034 | 0.008±0.0033 | 0.0864±0.0514 | **0.0137*** | **0.0262*** | 0.5053 | 0.8808 | 0.2038 | 0.4847 |
| *Actinomyces* | 0.0295±0.0124 | 0.0403±0.0076 | 0.0272±0.0059 | 0.0242±0.0071 | 0.0544 | 0.5888 | 0.7582 | 0.3720 | 0.2038 | 0.9248 |
| *Prevotella 7* | 0.0231±0.0091 | 0.0328±0.0111 | 0.0344±0.0095 | 0.0296±0.0078 | 0.8898 | 0.7477 | 0.3788 | 0.9546 | 0.8078 | 0.8049 |
| *Fusobacterium* | 0.025±0.0087 | 0.0296±0.0102 | 0.0246±0.0088 | 0.0394±0.0119 | 0.9699 | 0.7477 | 0.6121 | 0.7729 | 0.7537 | 0.4922 |
| *Atopobium* | 0.0219±0.0100 | 0.0536±0.0202 | 0.0242±0.0079 | 0.0146±0.0062 | 0.3007 | 0.8976 | 0.7653 | 0.5390 | 0.2038 | 0.8198 |
| *Akkermansia* | 0.0872±0.0211 | 0.0048±0.0035 | 0.0151±0.0070 | 0.0018±0.0014 | **0.0131*** | **0.0223*** | **0.0064**** | 0.7729 | 1.0000 | 0.6285 |
| *Porphyromonas* | 0.02±0.0079 | 0.0332±0.0121 | 0.0249±0.0071 | 0.0287±0.0086 | 0.9699 | 0.9604 | 0.5884 | 0.9546 | 0.8078 | 0.7207 |
| *Gemella* | 0.0337±0.0085 | 0.034±0.0123 | 0.0143±0.0040 | 0.0207±0.0060 | 0.9154 | 0.0562 | 0.3788 | 0.2263 | 0.4902 | 0.7207 |
| *Saccharimonadaceae(f)* | 0.0071±0.0042 | 0.0482±0.0198 | 0.019±0.0089 | 0.0181±0.0088 | **0.0211*** | 0.8023 | 0.9618 | 0.1409 | 0.2038 | 0.8509 |
| *Rothia* | 0.009±0.0019 | 0.0202±0.0073 | 0.0274±0.0066 | 0.0157±0.0045 | 0.9154 | 0.5888 | 0.9527 | 0.9546 | 0.8078 | 0.8049 |
| *Granulicatella* | 0.0036±0.0009 | 0.0282±0.0108 | 0.0194±0.0060 | 0.0151±0.0055 | **0.0211*** | 0.1907 | 0.4221 | 0.5654 | 0.4902 | 0.8509 |
| *Prevotella* | 0.0116±0.0046 | 0.0147±0.0066 | 0.016±0.0046 | 0.0233±0.0071 | 0.9154 | 0.9461 | 0.3544 | 0.9546 | 0.4902 | 0.4847 |
| *Ruminococcaceae UCG-014* | 0.0206±0.0046 | 0.0127±0.0067 | 0.0099±0.0036 | 0.0043±0.0019 | 0.3404 | 0.0970 | 0.0564 | 0.5654 | 0.4902 | 0.9250 |
| *Faecalibacterium* | 0.0018±0.0001 | 0.0306±0.024 | 0.0085±0.0032 | 0.0004±0.0002 | 0.8898 | 0.7477 | 0.5884 | 0.9546 | 0.4902 | 0.5916 |
| *Escherichia-Shigella* | 0.005±0.0017 | 0.0183±0.0098 | 0.0158±0.0062 | 0.0018±0.0017 | 0.6490 | 0.5888 | 0.0564 | 1.0000 | 0.4902 | 0.5916 |
| *Lachnospiraceae NK4A136 group* | 0.0336±0.0084 | 0.001±0.0007 | 0.0029±0.0013 | 0.0024±0.0016 | **0.0035*** | **0.0009*** | **0.0064*** | 0.9546 | 0.5959 | 0.8049 |
| *Alloprevotella* | 0.0047±0.0036 | 0.013±0.0069 | 0.0049±0.0018 | 0.0154±0.0059 | 0.3355 | 0.2824 | **0.0451*** | 0.9546 | 0.7046 | 0.4847 |
| *Acinetobacter* | 0.0193±0.0053 | 0.0005±0.0004 | 0.0071±0.0028 | 0.0044±0.0025 | **0.0035*** | **0.0031*** | **0.0064*** | 0.9546 | 1.0000 | 0.8509 |

^†^ Differences between groups were compared with Mann-Whitney U test with Benjamini & Hochberg correction. *q < 0.05; **< 0.01.
