# Supplementary material for: Dysbiotic change in gastric microbiome and its functional implication in gastric carcinogenesis
Source: Sci Rep. 2022 Mar 11;12:4285. doi: 10.1038/s41598-022-08288-9 (PMC8917121; doi:10.1038/s41598-022-08288-9)
Supplement: Supplementary file 7 — Supplementary Information 7. [file 41598_2022_8288_MOESM7_ESM.doc]

**Supplementary Table 3. Predicted functional pathways of gastric microbiome in GA and cancer groups based on KEGG database**

| **Pathways^†^** | **Gastritis** | | **AGC+EGC** | | **q-value** |
| --- | --- | --- | --- | --- | --- |
|  | **Mean** | **SE** | **Mean** | **SE** |  |
| Metabolic pathways | 0.1481 | 0.0005 | 0.1482 | 0.0007 | 0.5973 |
| Biosynthesis of secondary metabolites | 0.0644 | 0.0006 | 0.0628 | 0.0005 | 0.1283 |
| ABC transporters | 0.0563 | 0.0027 | 0.0559 | 0.0016 | 0.8762 |
| Biosynthesis of antibiotics | 0.0505 | 0.0004 | 0.0488 | 0.0002 | **0.0348*** |
| Microbial metabolism in diverse environments | 0.0450 | 0.0010 | 0.0435 | 0.0005 | 0.3465 |
| Biosynthesis of amino acids | 0.0292 | 0.0002 | 0.0286 | 0.0003 | 0.8329 |
| Two-component system | 0.0268 | 0.0006 | 0.0268 | 0.0010 | 0.2526 |
| Quorum sensing | 0.0263 | 0.0010 | 0.0258 | 0.0005 | 0.8762 |
| Carbon metabolism | 0.0243 | 0.0004 | 0.0245 | 0.0002 | 0.8329 |
| Purine metabolism | 0.0177 | 0.0006 | 0.0185 | 0.0003 | 0.4992 |
| Ribosome | 0.0171 | 0.0009 | 0.0188 | 0.0006 | 0.2073 |
| Pyrimidine metabolism | 0.0141 | 0.0007 | 0.0151 | 0.0003 | 0.4667 |
| Amino sugar and nucleotide sugar metabolism | 0.0132 | 0.0004 | 0.0131 | 0.0003 | 0.8762 |
| Glycolysis / Gluconeogenesis | 0.0117 | 0.0002 | 0.0112 | 0.0002 | 0.2617 |
| Pyruvate metabolism | 0.0114 | 0.0001 | 0.0114 | 0.0001 | 0.8329 |
| Phosphotransferase system (PTS) | 0.0114 | 0.0013 | 0.0113 | 0.0009 | 0.8762 |
| Starch and sucrose metabolism | 0.0108 | 0.0008 | 0.0101 | 0.0006 | 0.6440 |
| beta-Lactam resistance | 0.0069 | 0.0002 | 0.0060 | 0.0001 | **0.0348*** |

† Functional pathways with mean relative abundance > 1%, or those with 1% ≥ relative abundance > 0.5% and showing significant difference between
gastritis and the cancer group were listed. Differences between groups were compared with Mann-Whitney U test with Benjamini & Hochberg correction.
 *q < 0.05; **< 0.01.
