# Supplementary material for: Dysbiotic change in gastric microbiome and its functional implication in gastric carcinogenesis
Source: Sci Rep. 2022 Mar 11;12:4285. doi: 10.1038/s41598-022-08288-9 (PMC8917121; doi:10.1038/s41598-022-08288-9)
Supplement: Supplementary file 8 — Supplementary Information 8. [file 41598_2022_8288_MOESM8_ESM.doc]

**Supplementary Table 4. Predicted functional pathways of gastric microbiome in GA, ECG, and AGC groups based on KEGG database**

|  | **GA** | **EGC** | **AGC** | **q-value^†^** | | | |
| --- | --- | --- | --- | --- | --- | --- | --- |
| **Pathways** | **Mean ± SE** | **Mean ± SE** | **Mean ± SE** | **GA-EGC** | **GA-AGC** | **EGC-AGC** |  |
| Biosynthesis of secondary metabolites | 0.0644±0.0006 | 0.0630±0.0006 | 0.0625±0.0008 | 0.2372 | **0.0454*** | 0.7197 |  |
| Biosynthesis of antibiotics | 0.0505±0.0004 | 0.0492±0.0002 | 0.0481±0.0004 | 0.1743 | **0.0037**** | 0.1476 |  |
| Ribosome | 0.0171±0.0009 | 0.0183±0.0008 | 0.0198±0.0007 | 0.3923 | **0.0215*** | 0.1476 |  |
| Cysteine and methionine metabolism | 0.0091±0.0002 | 0.0097±0.0002 | 0.0099±0.0002 | 0.2021 | **0.0454*** | 0.7570 |  |
| Aminoacyl-tRNA biosynthesis | 0.0085±0.0004 | 0.0092±0.0004 | 0.0102±0.0004 | 0.3146 | **0.0069**** | 0.1476 |  |
| Glycine, serine and threonine metabolism | 0.0079±0.0001 | 0.0077±0.0001 | 0.0076±0.0001 | 0.2105 | **0.0254*** | 0.8190 |  |
| Homologous recombination | 0.0067±0.0004 | 0.0073±0.0003 | 0.0079±0.0003 | 0.2559 | **0.0183*** | 0.1476 |  |
| Bacterial secretion system | 0.0064±0.0002 | 0.0077±0.0004 | 0.0082±0.0006 | 0.1743 | **0.0457*** | 0.6962 |  |
| beta-Lactam resistance | 0.0069±0.0002 | 0.0061±0.0002 | 0.0057±0.0002 | 0.1743 | **0.0014**** | 0.2884 |  |
| Pentose phosphate pathway | 0.0059±0.0002 | 0.0062±0.0001 | 0.0063±0.0001 | 0.2105 | **0.0454*** | 0.3183 |  |
| Mismatch repair | 0.0055±0.0003 | 0.0060±0.0002 | 0.0066±0.0002 | 0.2372 | **0.0053**** | 0.1476 |  |
| DNA replication | 0.0050±0.0002 | 0.0053±0.0002 | 0.0057±0.0002 | 0.2678 | **0.0127*** | 0.1476 |  |
| Protein export | 0.0049±0.0002 | 0.0053±0.0002 | 0.0057±0.0002 | 0.3146 | **0.0358*** | 0.5476 |  |
| 2-Oxocarboxylic acid metabolism | 0.0053±0.0002 | 0.0049±0.0002 | 0.0042±0.0002 | 0.2455 | **0.0037**** | 0.1476 |  |

† Functional pathways with mean relative abundance > 1%, or those with 1% ≥ relative abundance > 0.5% and showing significant difference between
gastritis and AGC group were listed. Differences between groups were compared with Mann-Whitney U test with Benjamini & Hochberg correction.
*q < 0.05; **< 0.01.
